# Supplementary material for: Distinctive Properties of Endothelial Cells from Tumor and Normal Tissue in Human Breast Cancer
Source: Int J Mol Sci. 2021 Aug 17;22(16):8862. doi: 10.3390/ijms22168862 (PMC8396343; doi:10.3390/ijms22168862)
Supplement: Supplementary file 1 [file ijms-22-08862-s001.zip › ijms-1310691-SI.pdf]

Supplementary Data

S1 New cell lines express surface adhesion molecules

The levels of CD54 (ICAM-1) on the cells surface, were lower in tumor-derived ECs than in healthy ECs and the same tendency was found for CD31 (PECAM-1) (Figure S1). The cells show expression of VCAM-1 and were CD-62L+ with no significant differences between cell lines (Figure S1).

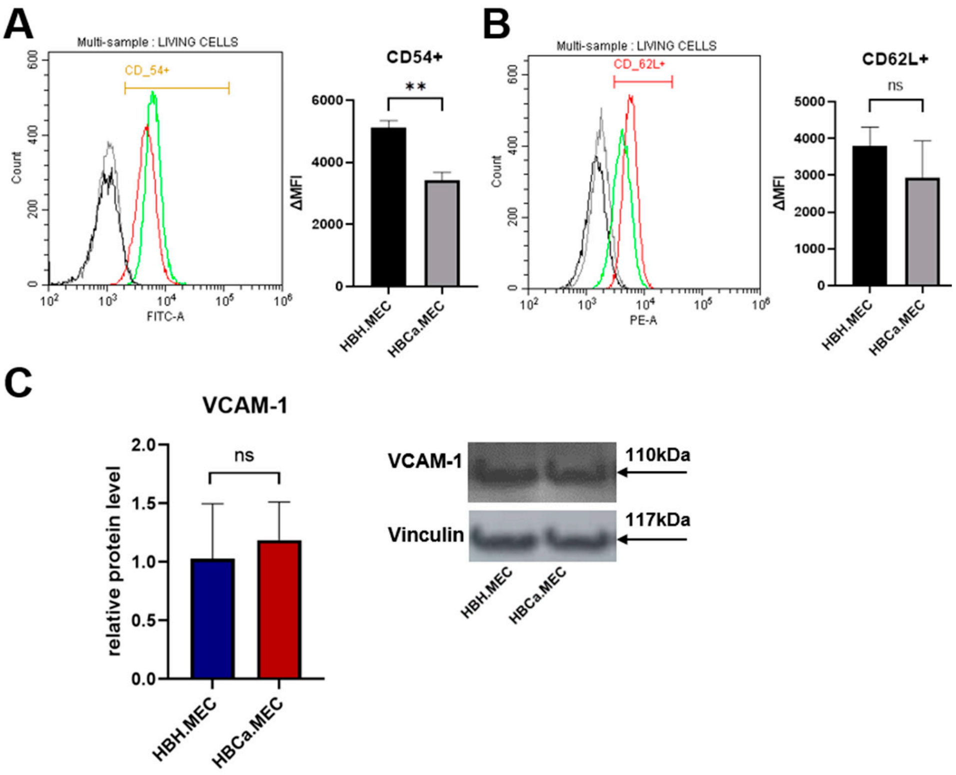

Figure S1. Differences in ECs phenotype on endothelial adhesion molecules ICAM-1 (CD54), VCAM-1 and CD62L. **A,B**- flow cytometric evaluation of CD54 and CD62-L expression. Data were recorded for 10,000 events using CellQuest software (v.2.3.0.84) and presented as histogram overlays (gray- healthy ECs unstained, black- tumor ECs unstained, green- healthy ECs stained, red- tumor ECs stained; on Y axis= the number of events; X axis= fluorescence intensity). Representative repetitions are shown (n = 3). The bar charts present delta MFI. ns- not significant; ** p <0.01 in Student t test *vs* HBH.MEC. Data are reported as the means ± SEM (n = 3). **C**-Relative protein level of VCAM-1 (110 kDa) for both cell lines. Loading control Vinculin (117 kDa). Representative bands are shown. Bar char presents data from ImageJ analysis (v.1.52p). Data are reported as the means ± SEM (n = 3). ns- not significant in Student t test vs HBH.MEC.

S2 Breast tumor-derived ECs display higher permeability markers than healthy breast ECs

The permeability of blood vessels is mainly controlled by ECs, therefore we have checked how the microenvironment influences on healthy and tumor-derived ECs leaking properties. Both cell lines were cultured on wells filled with filter upon normoxia. Healthy ECs exhibit lower permeability compared to pathological ECs (Figure S2A). Therefore, we evaluated the changes in tight junction protein, ZO-1 which displayed a lower level in pathological ECs *vs* healthy ECs (Figure S2B).

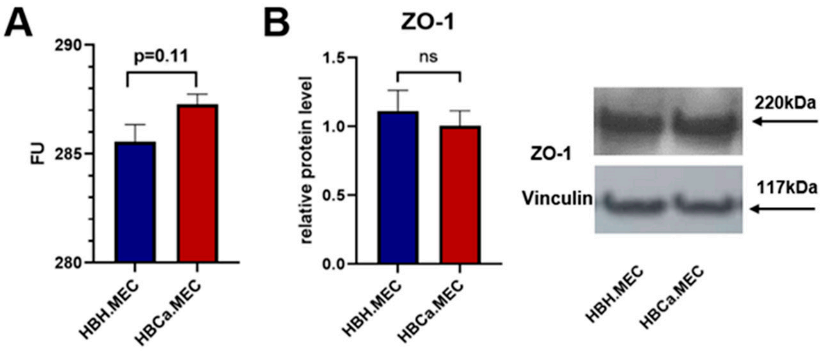

Figure S2. The influence of ECs phenotype on permeability. **A**- Fluorescence fold change of the medium after 48h culture of the cells in normoxia. **B**- Relative protein level of ZO-1 (220 kDa) for both cell lines. Loading control Vinculin (117 kDa). Representative bands are shown. Bar char presents data from ImageJ analysis (v.1.52p). Data are reported as the means ± SEM (n = 3). ns- not significant; p = 0.11 in Student t test vs HBH.MEC.
